# Prevalence and Clinical Consequences of Colistin Heteroresistance and Evolution into Full Resistance in Carbapenem-Resistant Acinetobacter baumannii

**DOI:** 10.1128/spectrum.05093-22

**Published:** 2023-05-23

**Authors:** Hadas Kon, Amichay Hameir, Amir Nutman, Elizabeth Temkin, Alona Keren Paz, Jonathan Lellouche, David Schwartz, David S. Weiss, Keith S. Kaye, George L. Daikos, Anna Skiada, Emanuele Durante-Mangoni, Yael Dishon Benattar, Dafna Yahav, Vered Daitch, Mariano Bernardo, Domenico Iossa, Lena E. Friberg, Ursula Theuretzbacher, Leonard Leibovici, Yaakov Dickstein, Dina Pollak, Sigal Mendelsohn, Mical Paul, Yehuda Carmeli

**Affiliations:** a National Institute for Antibiotic Resistance and Infection Control, Israel Ministry of Health, Tel Aviv, Israel; b Sackler School of Medicine, Tel Aviv University, Tel Aviv, Israel; c Adelson School of Medicine, Ariel University, Israel; d Emory Antibiotic Resistance Center, Emory University School of Medicine, Atlanta, Georgia, USA; e Robert Wood Johnson Medical School, Rutgers University, New Brunswick, New Jersey, USA; f First Department of Medicine, Laikon General Hospital, Athens, Greece; g National and Kapodistrian University of Athens, Athens, Greece; h Department of Precision Medicine, University of Campania Luigi Vanvitelli, Naples, Italy; i AORN dei Colli-Monaldi Hospital, Naples, Italy; j Institute of Infectious Diseases, Rambam Health Care Campus, Haifa, Israel; k Ruth and Bruce Rappaport Faculty of Medicine, Technion, Israel Institute of Technology, Haifa, Israel; l Infectious Diseases Unit, Rabin Medical Center, Beilinson Hospital, Petah Tikva, Israel; m Department of Medicine E, Rabin Medical Center, Beilinson Hospital, Petah Tikva, Israel; n Microbiology and Virology Unit, AORN Ospedali dei Colli-Monaldi Hospital, Naples, Italy; o Department of Pharmacy, Uppsala University, Uppsala, Sweden; p Center for Anti-Infective Agents, Vienna, Austria; q Microbiology Laboratory, Rambam Health Care Campus, Haifa, Israel; Rambam, Israel; Rambam, Israel; Rambam, Israel; Rambam, Israel; Rambam, Israel; Rambam, Israel; Rambam, Israel; Rambam, Israel; Rambam, Israel; Rambam, Israel; Rambam, Israel; Rambam, Israel; Rambam, Israel; Rambam, Israel; Beilinson, Israel; Beilinson, Israel; Beilinson, Israel; Beilinson, Israel; Beilinson, Israel; Beilinson, Israel; Beilinson, Israel; Beilinson, Israel; Sourasky, Israel; Sourasky, Israel; Sourasky, Israel; Sourasky, Israel; Greece, Laikon; Greece, Laikon; Greece, Laikon; Attikon, Greece; Attikon, Greece; Naples, Italy; Naples, Italy; Naples, Italy; Naples, Italy; Naples, Italy; Naples, Italy; Naples, Italy; Naples, Italy; Naples, Italy; Naples, Italy; Naples, Italy; Naples, Italy; Naples, Italy; Naples, Italy; The Netherlands; Sweden; Austria; Emory University School of Medicine

**Keywords:** *Acinetobacter baumannii*, carbapenem-resistance, colistin, heteroresistance, population analysis profiling

## Abstract

Colistin heteroresistance (HR) refers to a bacterial population comprised of several subpopulations with different levels of resistance to colistin. In this study, we discuss the classic form of HR, in which a resistant subpopulation exists within a predominantly susceptible population. We investigated the prevalence of colistin HR and its evolution into full resistance among 173 clinical carbapenem-resistant Acinetobacter baumannii isolates and examined the effect of HR on clinical outcomes. To determine HR, we performed population analysis profiling. Our results showed a high prevalence of HR (67.1%). To examine evolution of HR strains into full resistance, the HR strains were grown in colistin-containing broth, transferred onto colistin-containing plates, and colonies on these plates were transferred into colistin-free broth. Many of the HR strains (80.2%) evolved into full resistance, 17.2% reverted to HR, and 2.6% were borderline. We used logistic regression to compare 14-day clinical failure and 14-day mortality between patients infected by HR versus susceptible non-HR carbapenem-resistant A. baumannii. In the subgroup of patients with bacteremia, HR was significantly associated with 14-day mortality.

**IMPORTANCE** To our knowledge, this is the first large-scale study to report on HR in Gram-negative bacteria. We described the prevalence of colistin HR in a large sample of carbapenem-resistant A. baumannii isolates, the evolution of many colistin HR isolates to a resistant phenotype following colistin exposure and withdrawal, and the clinical consequences of colistin HR. We found a high prevalence of HR among clinical carbapenem-resistant A. baumannii isolates; most evolved into a resistant phenotype following colistin exposure and withdrawal. In patients treated with colistin, evolution of HR A. baumannii into full resistance could lead to higher rates of treatment failure and contribute to the reservoir of colistin-resistant pathogens in health care settings.

## INTRODUCTION

Carbapenem-resistant Acinetobacter baumannii (CRAB) is ranked as “critical” in the World Health Organization's list of priority pathogens for antibiotic development (1). Colistin is a last-line agent for treating infections caused by CRAB and other extremely drug-resistant Gram-negative pathogens. There are no additional approved antibiotics against CRAB except cefiderocol. However, CRAB-infected patients treated with cefiderocol have a higher case fatality rate than colistin-treated patients (2). Colistin remains the only treatment for most CRAB strains, but colistin treatment, even for susceptible strains, often fails. A randomized clinical trial (AIDA) comparing colistin monotherapy to colistin-meropenem for the treatment of severe carbapenem-resistant, colistin-susceptible infections, found that 14-day clinical failure was greater than 70% in both study arms (3).

HR is broadly defined as a bacterial population comprised of different subpopulations exhibiting various levels of susceptibilities to an antibiotic (4). In this study, we focused on the classic form of HR, in which a bacterial strain comprises a minority subpopulation of cells that exhibits increased levels of resistance compared to its predominantly susceptible population (4, 5).

Colistin HR has been reported in several bacterial species, including Enterobacter cloacae (6), Klebsiella pneumoniae (7), Stenotrophomonas maltophilia (8), and *Achromobacter* species (9). It was first described in A. baumannii in 2006 (10). HR goes unnoticed by clinical microbiology laboratories because the recommended method to determine colistin susceptibility, broth microdilution (BMD) (11), cannot detect HR. The gold standard for HR detection is population analysis profiling (PAP), which quantifies the proportion of resistant cells existing within a majority susceptible population using a high inoculum (12). Colistin resistance may occur following modifications in the lipid A, such as decreased negative charges due to removal or capping of phosphate residues, or alterations in the number, length, and hydroxylation status of the acyl chains (13, 14). In addition, in A. baumannii, colistin resistance may occur due to mutations in the genes involved in lipid A synthesis, resulting in lipopolysaccharide-defective strains (13). Colistin treatment for patients infected by HR bacteria may reduce or eliminate the majority colistin-susceptible population while the minority colistin-resistant subpopulation rapidly increases in size, potentially leading to treatment failure (15). The consequences of colistin HR in clinical settings have been described in case reports (16) but have not been examined in a large study.

In addition to studying HR, previous studies have examined the relationship between HR and resistance. Specifically, they examined whether an HR phenotype of a bacterial population is stable, i.e., if most of the population remains resistant once selection pressure is withdrawn (17). From a clinical perspective, following colistin treatment of an HR population, it is expected that only the colistin-resistant subpopulation will survive. Therefore, rather than focusing on the stability of the HR phenotype of a population, here, we focused on the resistant colonies (i.e., the resistant subpopulation) within the HR population and whether this subpopulation maintains their fully resistant phenotype after colistin pressure is removed, which we termed “evolution to full resistance.”

In this study, we investigated the prevalence of colistin HR, the evolution of the resistant subpopulation following colistin exposure and withdrawal, and the association between HR and clinical outcomes, using a large sample of CRAB isolates from the AIDA trial (3).

## RESULTS

### Sample.

Our sample consisted of 173 colistin-susceptible CRAB clinical isolates (meropenem MIC > 8 mg/L) from blood (*n* = 48) or other sites (*n* = 125). There were 171 index isolates (i.e., first isolate at infection onset) and 2 follow-up strains collected after colistin treatment.

The distribution of initial colistin MIC value (MIC-i) was as follows: 33 isolates (33/173, 19.1%) had an MIC-i of 0.5 mg/L, 115 (115/173, 66.5%) had an MIC-i of 1 mg/L, and 25 (25/173, 14.4%) had an MIC-i of 2 mg/L. Genetic typing revealed that 76 (76/173, 43.9%) belonged to clonal complex 2 (ST2), 69 isolates (69/173, 39.9%) to clonal complex 3 (ST3), and 28 (28/173, 16.2%) to other clonal complexes.

### HR prevalence.

**FIG 1:**
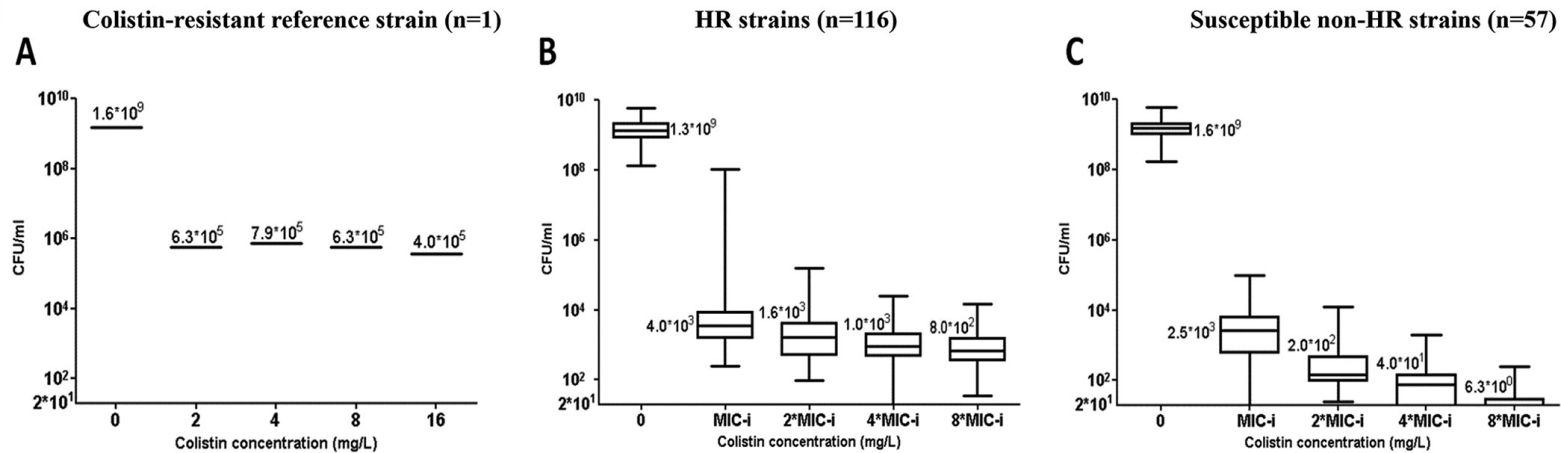
Population analysis profile of (A) a colistin-resistant reference strain, (B) HR strains and (C) susceptible non-HR strains. A to C show box plots of the number of colonies able to grow in the presence of each colistin concentration (CFU/mL). The horizontal line represents the average colony count (value presented outside the box), the box represents the upper and lower quartile and the vertical lines represent the highest and lowest value. Counts below 20 CFU/mL were considered insignificant (in figure C some points were not shown because the colony count was below the limit of 20 CFU/mL).

Fig. 1 shows the results of PAP in the unselected stage, by which 116 isolates (116/173, 67.1%) were classified as HR and 57 isolates (57/173, 32.9%) were classified as colistin susceptible non-HR. On plates without colistin, the average number of colonies was similar (*P* > 0.05) between the colistin-resistant reference isolate (Fig. 1A), the isolates that were ultimately categorized as HR (Fig. 1B), and the isolates that were ultimately categorized as colistin susceptible non-HR (Fig. 1C). The resistant reference isolate grew on colistin plates to an average count of 6.1*10^5^ CFU/mL (similar at all colistin concentrations tested). In the HR group and the susceptible non-HR group, the colony count decreased as the colistin concentration increased. At each colistin concentration tested, growth of HR strains was lower than the resistant reference isolate and higher than susceptible non-HR isolates (*P* < 0.001 for HR versus susceptible non-HR strains at all colistin concentrations). At 8*MIC-i colistin, the resistant reference isolate reached a count of 4.0*10^5^ CFU/mL, HR isolates grew to an average of 8.0*10^2^ CFU/mL, while the susceptible non-HR isolates grew to an insignificant count of 6.3 CFU/mL on average.

For the resistant reference isolate, the frequency of resistant cells in the unselected stage (F_U_) remained in the range of 2.5*10^−4^ to 4.9*10^−4^, while for the HR and susceptible non-HR isolates, F_U_ decreased as the colistin concentration increased. At MIC-i, the average F_U_ was similar between the HR group and the susceptible non-HR group (1.6*10^−6^ versus 3.1*10^−6^). However, at higher colistin concentrations, the F_U_ of the susceptible non-HR isolates decreased remarkably compared to the HR isolates. The 116 HR isolates were categorized as HR because growth was observed at 8*MIC-i (an average of 8.0*10^2^ CFU/mL) and their F_U_ at 8*MIC-i (6.1*10^−7^) was above the cutoff defining HR (10^−7^). The ATCC 19606 control strain was categorized as HR (growth on 8*MIC-i was 2.8*10^3^ CFU/mL, F_U_ 1.7*10^−6^). Of the 57 susceptible non-HR isolates, 23 did not grow on 8*MIC-i plates and 34 grew below the cutoff defining HR (3.9*10^−9^).

The prevalence of HR did not differ according to the initial MIC: for MIC-i of 0.5, 1, and 2 mg/L, HR prevalence was 63.6% (21/33), 69.6% (80/115), and 60.0% (15/25), respectively (*P* = 0.57). Additionally, no association was found between HR prevalence and clonal complex types.

### Evolution to full resistance.

**FIG 2:**
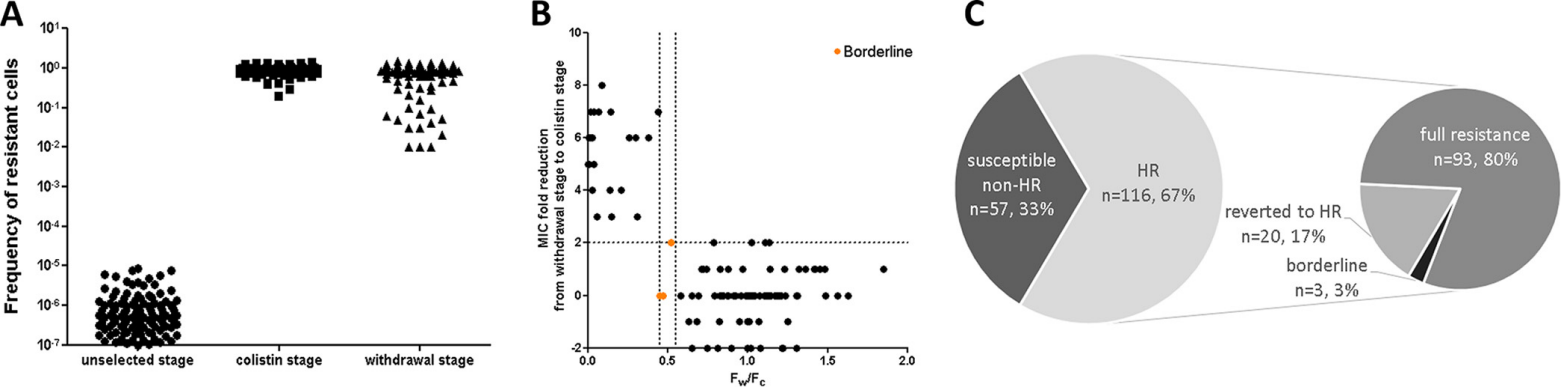
Panels A and B refer to HR isolates only. Panel C represents the entire study sample. (A) Frequencies of resistant cells of the HR isolates from the unselected stage (F_U_), colistin stage (F_C_) and withdrawal stage (F_W_) at 8*MIC-i. (B) Determination of evolution into full resistance or reversion into HR. Bottom right quarter are fully resistant and top left quarter are reverted. Three borderline isolates marked in orange. (C) Distribution of HR and susceptible non-HR isolates, including distribution of evolution into full resistance or reverted to HR.

The frequency of the resistant subpopulation among the HR isolates at the unselected stage, the colistin stage, and the withdrawal stage (F_U_, F_C_, and F_W_, respectively) at 8*MIC-i is presented in Fig. 2A. Unselected, the frequency of the resistant population (F_U_) ranged from 10^−7^ to 10^−5^. Exposure to colistin resulted in a markedly increased frequency of the resistant subpopulation (F_C_ ranging between 0.2 and ~1); i.e., in the colistin stage (following selection), the resistant subpopulation of the HR isolates became the majority population. In the withdrawal stage, in which the HR strains were subjected to three passages in colistin-free broth, the frequency of the resistant subpopulation varied between strains more than in the colistin stage (F_W_ ranging between 0.01 to ~1) but was much higher than in the unselected stage.

To determine whether the HR strains in the withdrawal stage evolved into full resistance or revert to HR (i.e., reverted), two factors were examined: the F_W_/F_C_ ratio and the MIC fold reduction from withdrawal stage (MIC_w_) to the colistin stage (MIC_c_). Almost all HR isolates could be fit into two groups: the “full resistance” in the bottom right quarter, and the “reverted” in the top left quarter (Fig. 2B). Of the HR isolates, 80.2% (93/116) evolved into full resistance, whereas 17.2% (20/116) reverted to HR. ATCC 19606 was included in the “reverted” group. Three strains had a borderline HR population after withdrawal. To summarize, Fig. 2C shows the distribution of strains of the entire sample.

### Association between HR and clinical outcomes.

Clinical data were available for 168/171 patients. After *in vitro* colistin exposure and withdrawal, the index isolates of 57 patients were susceptible non-HR, 23 reverted to HR (or were borderline), and 88 evolved into full resistance. Patients' demographic and medical characteristics are presented in Table 1. The association between HR phenotype and clinical outcomes is shown in Table 2. HR type was not significantly associated with 14-day clinical failure or 14-day mortality. However, in the subgroup of patients with bloodstream infection (BSI), the odds of 14-day mortality doubled (adjusted odds ratio [OR]: 2.3, 95% CI: 1.04, 5.06) as the HR type increased one level (from susceptible non-HR to reverted, and from reverted to full resistance). No differences were found between the treatment arms.

**TABLE 1:** Patient characteristics by heteroresistance (HR) phenotype of the index strains

Characteristics	Susceptible non-HR *(n = *57)	Reverted or borderline HR (*n* = 23)	Full resistance HR (*n* = 88)
Age, yrs, median (IQR)^*a*^	68 (54.5–79)	67 (43–80)	68.5 (56–79)
Sex, male, *n* (%)	31 (54.4)	10 (43.5)	68 (77.3)
Charlson score, median (IQR)	2 (1–3.5)	1 (0–4)	2 (0–3)
SOFA score, median (IQR)	7 (5–9)	6 (4–7)	6 (4–8)
Infection type			
Bacteremia, *n* (%)	21 (36.8)	4 (17.4)	22 (25)
Other, *n* (%)	36 (63.2)	19 (82.6)	66 (75)
Acquired in intensive care unit, *n* (%)	27 (47.4)	11 (47.8)	32 (36.4)
Treated with colistin-meropenem, *n* (%)	28 (49.1)	12 (52.2)	45 (51.1)

a IQR,interquartile range.

**TABLE 2:** Clinical outcomes by heteroresistance (HR) phenotype of the index strain^*a*^

Outcome	Type of infection	Susceptible non-HR *n*/N (%)	Reverted to HR/borderline *n*/N (%)	Full resistance *n*/N (%)	OR (95% CI)^*b*^	Adjusted OR (95% CI)^*c*^
14-day clinical failure	All (*n* = 168)	45/57 (78.9)	16/23 (69.6)	74/88 (84.1)	1.20 (0.79, 1.82)	1.35 (0.85, 2.15)
	BSI (*n* = 47)	16/21 (76.2)	2/4 (50.0)	19/22 (86.4)	1.36 (0.65, 2.87)	1.72 (0.71, 4.16)
14-day mortality	All (*n* = 168)	27/57 (47.4)	2/23 (8.7)	33/88 (37.5)	0.85 (0.6, 1.19)	0.9 (0.62, 1.3)
	BSI (*n* = 47)	4/21 (19.0)	1/4 (25.0)	10/22 (45.5)	1.89 (0.95, 3.78)	2.3 (1.04, 5.06)

a BSI, bloodstream infection; OR, odds ratio.

b HR was defined as an ordinal variable with 0 = susceptible non-HR, 1 = reverted to HR or borderline, and 2 = full resistance.

c Adjusted for age, Charlson score, SOFA score, and treatment arm.

## DISCUSSION

We found a high prevalence (67.1%) of HR among colistin-susceptible CRAB isolates causing severe infections. HR prevalence was independent of the isolate's initial colistin MIC. Before colistin exposure, the colistin-resistant minority population growing at 8*MIC-i comprised 1 of every 1 million bacterial cells. The importance of this minority population became evident upon exposure to colistin pressure; the frequency of the resistant population among the HR strain reached 20 to 100% of the entire bacterial population. Importantly, upon withdrawal of colistin pressure, the frequency of the resistant population remained much higher than the frequency among the unselected population.

The prevalence of colistin HR in A. baumannii in clinical settings varies between studies. Several studies reported high prevalence of HR (83 to 100%) (10, 16, 18–20), some found a slightly lower prevalence of 43 to 46% (21, 22), while others reported a much lower prevalence, between 0 and 23% (23–27). There are several possible explanations for the diverse results. First, they may reflect differences in the strains studied: their genotypes and mechanisms of resistance; the selection pressure that the isolates or their ancestors were exposed to before isolation; and study time, location, and isolate source (27). Second, studies differed in their HR detection methods. While the PAP assay is the gold standard for HR determination, some studies applied other methods, such as agar screen and Etest (17, 28). Third, while colistin HR is defined as a colistin-susceptible isolate in which a detectable subpopulation can grow in the presence of *x*-fold higher than the MIC of the susceptible majority, the value of *x* varied among studies. In our study, *x* was set at 8-fold MIC, as suggested previously (17, 18, 29), to ensure a substantial difference in MIC between the resistant subpopulation and the main susceptible population. However, in other studies, *x* was 2-fold, 4-fold, or higher than 8-fold (10, 19, 20, 22, 24, 26). If we had set *x* at 4-fold the initial MIC, the prevalence of HR would have been 71.7% (124/173).

We determined the evolution of the HR population following colistin selection and withdrawal. We found two distinct groups: those that evolved into a fully resistant population (80.2%), and those that reverted to HR (17.2%). These findings imply that the great majority of HR isolates evolve into resistant isolates after colistin treatment and will become the reservoir for spread of colistin resistance.

**FIG 3:**
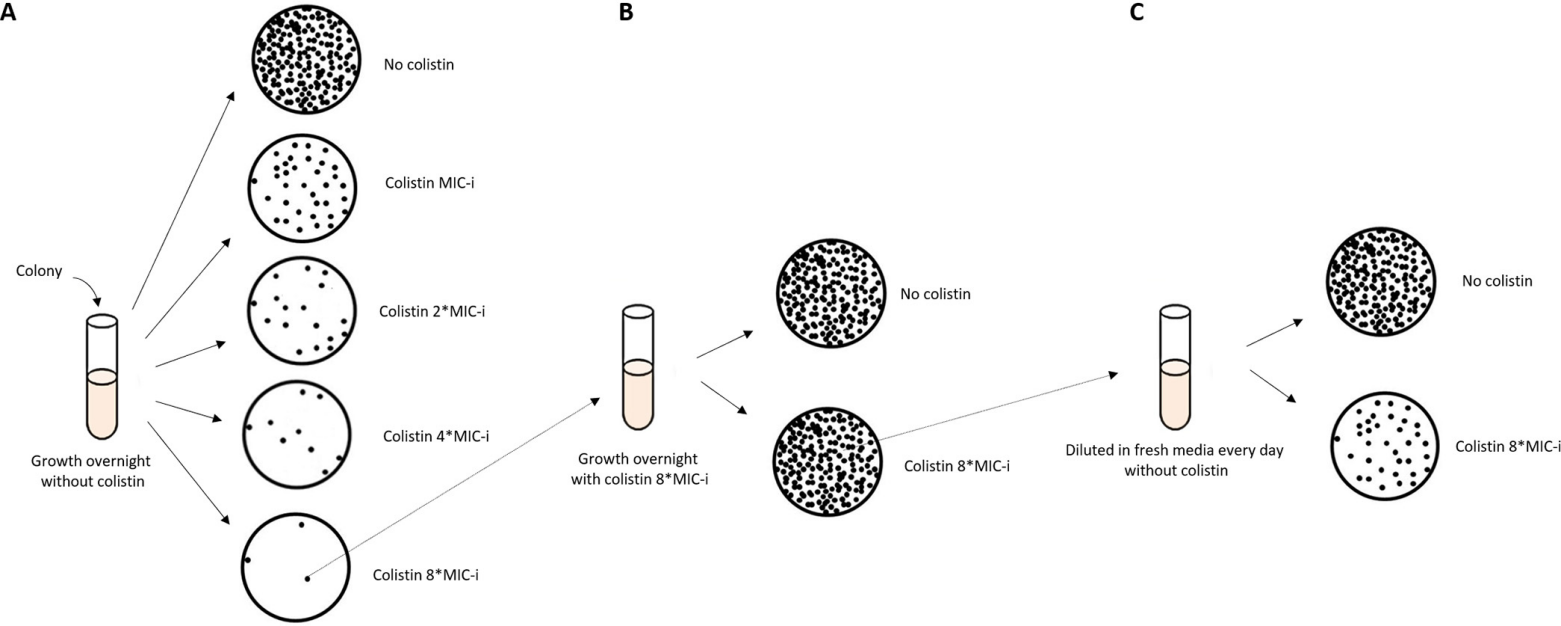
Schematic illustration of experiments conducted in this study to determine heteroresistance (HR) and evolution to full resistance: (A) Population analysis profiling (PAP) assay (unselected stage), (B) Selection of the resistant population (colistin stage) and (C) withdrawal of antibiotic pressure (withdrawal stage).

Our study aimed to detect evolution from HR to full resistance, whereas previous studies focused on detection of HR stability. While both study types use methods based on colistin selection and withdrawal of selection, they differ in the level of selection. Stability testing (10, 17, 20, 26) involves one round of colistin selection (i.e., overnight growth in broth containing colistin, as in Fig. 3B) and then withdrawal of selection pressure by transferring the enriched HR population into colistin-free broth. When testing evolution to full resistance, after the selection stage there is an additional step using only the colonies growing on the 8*MIC-i colistin-containing plate. By this extra step, only the homogenous subpopulation that can grow on 8*MIC-i colistin is transferred to the withdrawal stage. What are these two methods testing? Stability testing asks, within an HR population in which the minority resistant subpopulation was enriched by selection pressure, will the HR of the population be maintained once selection pressure is withdrawn? Our method asks, within an HR population in which the minority resistant subpopulation was enriched by selection pressure, will the resistant subpopulation selected maintain its fully resistant phenotype once selection pressure is withdrawn? The two questions have different implications. Stability is interesting from a biological point of view, shedding light on population dynamics under selection pressure and its withdrawal and hinting at reversible versus stable mechanisms of adaptation. Our method has epidemiological and clinical ramifications. The epidemiological significance is that evolution into full resistance, and its persistence during and after withdrawal of colistin treatment, creates an expanded reservoir of resistant strains that may spread between patients. The clinical significance is that evolution into full resistance means that resistance will persist during colistin therapy and potentially result in treatment failure.

The relationship between stability and evolution into full resistance is unknown. Future studies should aim to bridge between the biological questions and the epidemiological and clinical questions by examining whether a stable HR population is also more likely to evolve into full resistance, whether these are parallel phenomena, or whether only a subpopulation within a stable HR population has the potential to evolve into full resistance.

We examined the effect of HR in A. baumannii on patient outcomes. Our findings were intriguing: each increase in HR phenotype (from susceptible non-HR to reverted, and from reverted to full resistance) doubled the odds of 14-day mortality in patients with bacteremia, but not in other types of infections. There are several possible explanations: (i) This analysis pertained to a secondary aim of AIDA and was likely underpowered. (ii) AIDA included severely ill patients and overall mortality was very high. It is possible that competing factors leading to death negated the differences between HR phenotypes. (iii) HR may play a different role at different sites of infection, either because of differences in overall bacterial burden, differences in colistin concentration at different anatomic sites, or slower microbiological diagnosis and delayed treatment at sites other than blood. In the one previous study that examined the association between HR and clinical outcomes using a small sample of 24 patients, results were not statistically significant. Srinivas et al. studied patients with pneumonia or bacteremia caused by A. baumannii (18). Infection-related deaths occurred in 4/20 (20%) patients whose isolates were HR and 1/4 (25%) patients whose isolates were colistin susceptible non-HR (16). In our study, when comparing susceptible non-HR to HR, the association between HR and clinical outcomes was not statistically significant. We believe that this finding may show that differentiating between HR types (full resistance and reverted to HR) is important.

Our study has several limitations. First, various mechanisms may lead to HR, and their effect on clinical outcomes may differ. It was beyond the scope of this study to examine HR mechanisms. Second, colistin resistance has been linked to fitness cost; it may be that HR also has a fitness cost, but we did not examine this. Further studies using a larger sample and examining the mechanisms of HR and strain fitness are required to better understand the association between HR and patient outcomes. Third, in this study one resistant mutant from growth in the presence of colistin was selected for testing the evolution to full resistance. We did not study multiple colonies per patient. Thus, we cannot exclude the possibility that various mutants, which may behave differently, exist within the resistant strains. However, the large sample of isolates from which resistant colonies were randomly selected strengthens our confidence in the results.

In conclusion, in this first large-scale study of colistin HR in CRAB isolates using the gold standard method, we found alarming rates of HR. We found that most HR strains evolved into full resistance after colistin exposure and withdrawal, thus contributing to the colistin resistance reservoir and suggesting that treatment is likely to fail. Simple methods for incorporating HR detection into clinical laboratory testing are needed.

## MATERIALS AND METHODS

### Isolates.

AIDA was a multicenter randomized controlled trial (RCT) comparing colistin monotherapy to colistin-meropenem combination therapy for the treatment of severe carbapenem-resistant Gram-negative infections. The trial took place from October 2013 to December 2016 in six hospitals in Italy, Greece, and Israel. AIDA enrolled inpatients aged ≥18 years with hospital-acquired pneumonia (HAP), ventilator-associated pneumonia (VAP), bloodstream infection (BSI), or urinary tract infection (UTI) caused by carbapenem nonsusceptible A. baumannii, *Enterobacterales*, and Pseudomonas aeruginosa. Isolates were shipped to the central laboratory after subculturing and storage at −80°C. After isolation, none of the isolates were exposed to colistin before HR was analyzed. Of the 266 A. baumannii isolates sent to the central laboratory, 93 were colistin-resistant and therefore excluded from our study. The isolates used in this study were stored at −80°C for 5 years before HR testing.

All isolates were identified to the species level by Vitek MS (bioMérieux SA, Marcy l'Etoile, France). Species confirmation was conducted by molecular genotyping, as described previously (30). In brief, typing of A. baumannii isolates was performed by the alignment of OXA-51-like β-lactamase sequence relationships using OXA-69A and OXA-69B primers. Clonality was determined by sequencing of the OXA-51-like gene to assign isolates to international clonal complexes (Pasteur scheme). All isolates were stored at −80°C and subcultured twice on blood agar (Trypticase soy aga [TSA] + 5% blood sheep agar, HyLabs, Rehovot, Israel) at 35 ± 2°C before further testing.

### Meropenem and colistin susceptibility testing.

Meropenem MICs were determined using a home-made BMD assay according to ISO guidelines (31) and confirmed by a commercial BMD method (customized Sensititre plates, ThermoFisher Scientific, Oakwood Village, OH), at meropenem concentrations of 0.5 to 64 mg/L. Quality control strains were tested as required (32). Susceptibilities were determined using EUCAST 2021 breakpoints (susceptible ≤2 mg/L, resistant >8 mg/L) (33). When the two methods yielded discrepant results, both tests were repeated, and we used the predominant value as the final MIC.

Colistin susceptibility was determined using a home-made BMD assay in cation-adjusted Mueller-Hinton (CAMH) broth (Becton, Dickinson [BD], MD, USA) at colistin concentrations of 0.5 to 64 mg/L, according to ISO guidelines (31) and the recommendations of the CLSI-EUCAST Polymyxin Breakpoints Working Group (11). The test was performed in duplicates from two different suspensions, prepared from two distinct plates of the same isolate. MIC values were read using ISO guidelines (31) and susceptibilities were interpreted using EUCAST 2021 breakpoints (susceptible breakpoint of ≤2 mg/L) (33). Quality control strains were tested as required (32). Because the strains have been stored at −80°C, a third confirmation of colistin MIC was performed prior to the experiments.

### PAP assay.

To test for the presence of HR, the PAP assay was conducted on isolates at the unselected stage, i.e., following overnight growth without colistin (12). The PAP assay is illustrated in Fig. 3A. Colistin stock solution (1,000 mg/L) was prepared by resuspending lyophilized powder of colistin sulfate salt (Sigma-Aldrich, Merck KGaA, Darmstadt, Germany) in sterile water (molecular grade; Bio-Lab, Jerusalem, Israel). The colistin stock was diluted and added to CAMH agar (BD) to produce plates with colistin concentrations of 1, 2, 4, and 8 times the MIC-i of the tested isolate.

An isolated colony was inoculated into 2 mL sterile CAMH broth and incubated overnight (18 ± 2 h) at 34 to 37°C with shaking (200 rpm). Following a 10-fold serial dilution in sterile phosphate-buffered saline (Bio-Lab), a 100-μL aliquot was plated in duplicate (in two different dilutions) on each of the four plates containing colistin (1*, 2*, 4*, and 8* MIC) and on a CAMH colistin-free plate. Colonies were counted manually following incubation overnight. Due to the limitations of the spread plate technique, counts below 20 CFU/mL were considered insignificant, as shown previously (24, 34). For the statistical analysis, we used the mean of the colony counts from the four plates for each isolate at each colistin concentration (two different dilutions in duplicate).

The frequency of resistant cells at each colistin concentration was defined as the CFU that grew on plates containing colistin (i.e., the resistant subpopulation) divided by the CFU that grew on colistin-free plates (i.e., the total CFU of the isolate) (17). The frequency at the unselected stage (F_U_) was calculated from the PAP assay. Strains were classified as HR if growth of A. baumannii colonies was observed on plates containing colistin at a concentration 8-fold higher than the MIC of the susceptible main population (8*MIC-i) with a frequency (F_U_) of >10^−7^ at 8*MIC-i (17, 18, 29). A colistin-resistant A. baumannii strain with mutations in *pmrB* (A138T) and *lpxD* (E117K) with a known colistin MIC of 32 mg/L was used as a reference strain and the colistin-susceptible ATCC 19606 (MIC 1 mg/L) was used as the control.

### Evolution of the resistant subpopulation into full resistance.

We conducted a two-stage process for the HR strains only, to examine whether the HR population within a given isolate exposed to colistin reverts to a susceptible majority after the withdrawal of colistin or evolves into a fully resistant population. The first stage was the colistin stage (Fig. 3B). A single colony picked from the 8*MIC-i plate of the PAP assay was resuspended in 2 mL of CAMH broth containing colistin 8*MIC-i and grown overnight with shaking. Following incubation, bacterial dilutions were streaked in duplicate on CAMH plates containing colistin 8*MIC-i and on CAMH colistin-free plates. Colonies were counted after overnight incubation. The frequency of resistant cells at the colistin stage (F_C_) was calculated as described above and colistin MIC at the colistin stage (MIC_c_) was determined using 3 to 5 individual colonies growing on 8*MIC-i colistin plates.

The second stage was the withdrawal stage (Fig. 3C). A single colony picked from the 8*MIC-i plate of the colistin stage was grown overnight in 2 mL colistin-free CAMH broth with shaking (day 1). A dilution of 1:1,000 with fresh colistin-free CAMH broth was performed every day for two additional days. On day 4, the bacterial dilutions were streaked in duplicate on CAMH plates with colistin 8*MIC-i and on CAMH colistin-free plates and incubated overnight. The frequency of resistant cells at the withdrawal stage (F_W_) was calculated and colistin MIC at the withdrawal stage (MIC_w_) was determined using 3 to 5 individual colonies growing on colistin-free plates.

A strain was considered to have evolved into full resistance if the frequency of the resistant population did not change significantly between the withdrawal stage and the colistin stage (F_W_/F_C_>0.55), and the MIC_w_ was similar to the MIC_c_ (i.e., less than 2-fold dilutions different). A strain was considered to have reverted to HR if the frequency of the resistant population decreased significantly between the withdrawal stage and the colistin stage (F_W_/F_C_<0.45), and the MIC_w_ was more than 2-fold dilutions lower than the MIC_c_. A strain was considered borderline if F_W_/F_C_ was between 0.45 and 0.55. ATCC 19606 was used as a control.

### Association between HR and clinical outcomes.

We examined the effect of HR on two clinical outcomes: 14-day clinical failure (as defined in the AIDA trial [3]) and 14-day all-cause mortality. We also examined potential confounders that were collected in AIDA, including demographic characteristics, Charlson comorbidity score, sepsis severity (SOFA score), infection type, and study arm.

### Statistical analysis.

We used chi-square to test the association between initial colistin MIC and the prevalence of HR. Differences between mean colony counts were tested using Student's *t* test. We performed simple logistic regression to test the association between HR and clinical outcomes. HR was modeled as an ordinal variable (0 = susceptible non-HR, 1= reverted to HR or borderline HR, and 2 = full resistance) according to the PAP of the index isolate. We performed a multivariable logistic regression that included the covariates age, Charlson score, SOFA score, and study arm, selected *a priori*. We analyzed each outcome separately in all patients and in the subgroup of patients with BSI. Analyses were performed using SPSS version 27 (IBM Corporation, Armonk, NY).

### Ethics statement.

The trial was approved by the Institutional Review Boards of all participating hospitals.

### Data availability.

The AIDA data regarding patient characteristics and outcomes are available from the corresponding author upon reasonable request.
